# Posterior percutaneous fixation without anterior or posterior debridement for infectious spondylitis in high-morbidity patients: A retrospective comparative study

**DOI:** 10.1097/MD.0000000000046700

**Published:** 2026-01-02

**Authors:** Chan Young Lee, Sung-Kyu Kim, Hyoung-Yeon Seo

**Affiliations:** a Department of Orthopedic Surgery, Chonnam National University Hwasun Hospital, Hwasun-gun, Jeollanam-do, Republic of Korea; b Department of Orthopedic Surgery, Chonnam National University Medical School and Hospital, Gwang-ju, Republic of Korea.

**Keywords:** high morbidity, infectious spondylitis, minimally invasive spine surgery, percutaneous pedicle screw fixation

## Abstract

Standard surgical treatment for infectious spondylitis involves anterior debridement and interbody fusion. However, for patients with high morbidity, this may result in many complications. We propose an alternative technique – posterior-only percutaneous pedicle screw fixation without anterior or posterior debridement (“internal orthosis”) – to reduce surgical invasiveness while maintaining clinical efficacy. We retrospectively compare the outcomes between conventional and internal orthosis procedures. This retrospective comparative study included patients who underwent surgery for thoracolumbar infectious spondylitis between 2012 and 2021. Patients were divided into 2 groups according to surgical technique: conventional treatment with anterior debridement and posterior fixation, and posterior-only fixation without any debridement. Clinical outcomes (visual analog scale [VAS] score and success rate), laboratory markers (white blood cell count, erythrocyte sedimentation rate, and C-reactive protein), and radiologic parameters (kyphotic/lordotic angle and bone union) were evaluated during follow-up. A total of 45 patients were included in the study (22 in the conventional group and 23 in the internal orthosis group). The internal orthosis group demonstrated significantly shorter operative time (202 ± 71 minutes vs 376 ± 126 minutes, *P* < .001) and reduced intraoperative blood loss (267 ± 122 mL vs 646 ± 460 mL, *P* < .001) compared with the conventional group. Inflammatory markers and VAS score improved significantly over time in both groups. Radiologic analysis showed a transient increase in kyphotic angle in the internal orthosis group at one month, although long-term spinal alignment remained stable in both groups. While the conventional group demonstrated a higher rate of radiologic bone union (86.4% vs 43.5%, *P* = .003), the internal orthosis group achieved similar clinical success, with rates of 95.5% and 87.0%, respectively (*P* = .608). No other significant group differences were identified in laboratory or radiologic parameters. “Internal orthosis” can be an alternative treatment option for high-risk patients with infectious spondylitis. Despite a lower rate of radiological fusion, clinical outcomes were comparable, suggesting its potential as a truly minimally invasive strategy in selected cases.

## 1. Introduction

Infectious spondylitis is a rare but serious condition with an increasing incidence, reported as 2.4 to 6.5 cases per 100,000 persons annually, and is associated with considerable morbidity and mortality.^[1,2]^ Recent epidemiologic analyses have demonstrated that the in-hospital mortality rate ranges from 1.8 to 5.5% within one year, and may reach up to 45% at long-term follow-up, highlighting the substantial clinical burden of this disease.^[3]^ Despite advances in antibiotics and surgical techniques, controversy persists regarding the optimal surgical strategy for high-risk patients.^[4–6]^

In infectious spondylitis, the anterior column is most commonly affected; therefore, anterior debridement with tricortical bone strut graft or titanium cage instrumentation has been considered the gold standard treatment.^[7,8]^ Debridement of the necrotic portion of the vertebra is an essential principle in musculoskeletal infection surgery. However, this procedure is technically demanding and results in many complications. Moreover, because major vessels such as the vena cava, aorta, and azygos system are located around the anterior column, anterior debridement can further increase surgical morbidity. In patients with high operative risk – such as elderly individuals or those with poor general condition and multiple comorbidities – such invasive procedures may increase the likelihood of perioperative complications and morbidity.^[9,10]^

Recent literature has explored less invasive alternatives. Yunoki et al reviewed the management of pyogenic spondylitis and highlighted the growing role of minimally invasive approaches in patients with severe comorbidities.^[2]^ Extending this concept, McLean et al demonstrated that treatment strategies are often determined by the severity and anatomical extent of infection, suggesting the importance of individualized surgical decision-making based on disease stage rather than a uniform approach.^[3]^

To reduce surgical invasiveness, several studies have reported favorable outcomes with posterior-only approaches, including posterior debridement and interbody fusion with instrumentation.^[11–13]^ Building on this concept, we further minimized surgical invasiveness by posterior-only percutaneous fixation technique without anterior or posterior debridement and interbody fusion – a method we refer to as an “internal orthosis.” By providing spinal stability through instrumentation and achieving infection control via systemic antibiotic therapy alone, we hypothesized that this technique could serve as a safer and less invasive surgical option for patients with high morbidity. To evaluate its efficacy, we conducted a retrospective comparison between this method and the conventional anterior-posterior approach.

## 2. Material & method

### 2.1. Patients

We retrospectively reviewed the medical records of patients who underwent surgical treatment for thoracolumbar infectious spondylitis at Chonnam National University Hospital between 2012 and 2021. Inclusion criteria were as follows:

1. Received at least 3 weeks of antibiotic therapy for infectious spondylitis
2. Among these, patients were considered to have failed conservative treatment if they met one or more of the following conditions^[14]^:
  A. Persistent severe back pain (visual analog scale [VAS] ≥ 7), to the extent that the patient was unable to sit
  B. Failure of normalization of inflammatory markers (white blood cell count [WBC], erythrocyte sedimentation rate [ESR], and C-reactive protein [CRP])

Exclusion criteria included:

1. Cases in which surgery was performed before the completion of 3 weeks of antibiotic therapy due to complications (neurologic compromise, sepsis, etc)
2. Cases in which corpectomy or vertebral body reconstruction procedures
3. Follow-up duration of <6 months

Patients who met the inclusion criteria and did not meet any exclusion criteria were included in the study. They were subsequently divided into 2 groups according to the surgical method: the anterior debridement with percutaneous pedicle screw fixation group (conventional group) and the posterior-only percutaneous pedicle screw fixation group (internal orthosis group).

### 2.2. Surgical technique

The choice of surgical procedure was determined by the patient’s overall condition, comorbidities, and the extent of infection. Posterior-only percutaneous fixation without debridement (“internal orthosis”) was first performed in 2014 for high-morbidity patients who could not tolerate anterior debridement. Over time, this technique was increasingly applied to patients with limited infection extension – those without severe bone destruction or large anterior paravertebral abscesses – regardless of their general condition, as part of a minimally invasive surgical strategy.

In the conventional group, the patient was placed in the lateral decubitus position. After discectomy, anterior interbody fusion was performed using a tricortical iliac autogenous bone graft. After the anterior procedure, the patient was placed in the prone position, and percutaneous pedicle screw fixation was performed on the involved and adjacent vertebrae. If the bone quality was poor, additional segments were instrumented to ensure stable fixation.

In the internal orthosis group, only the posterior procedure was performed in the prone position. Percutaneous pedicle screw fixation was performed in the same manner as in the conventional group. If preoperative magnetic resonance imaging or computed tomography showed an epidural or paravertebral abscess, posterior decompression or drainage was performed as necessary.

Two board-certified spine surgeons performed all procedures.

Postoperatively, all patients wore a thoracolumbosacral orthosis and were encouraged to ambulate with assistive devices. Intravenous antibiotics were administered for at least 6 weeks after surgery.

### 2.3. Data collection

**Figure 1.**
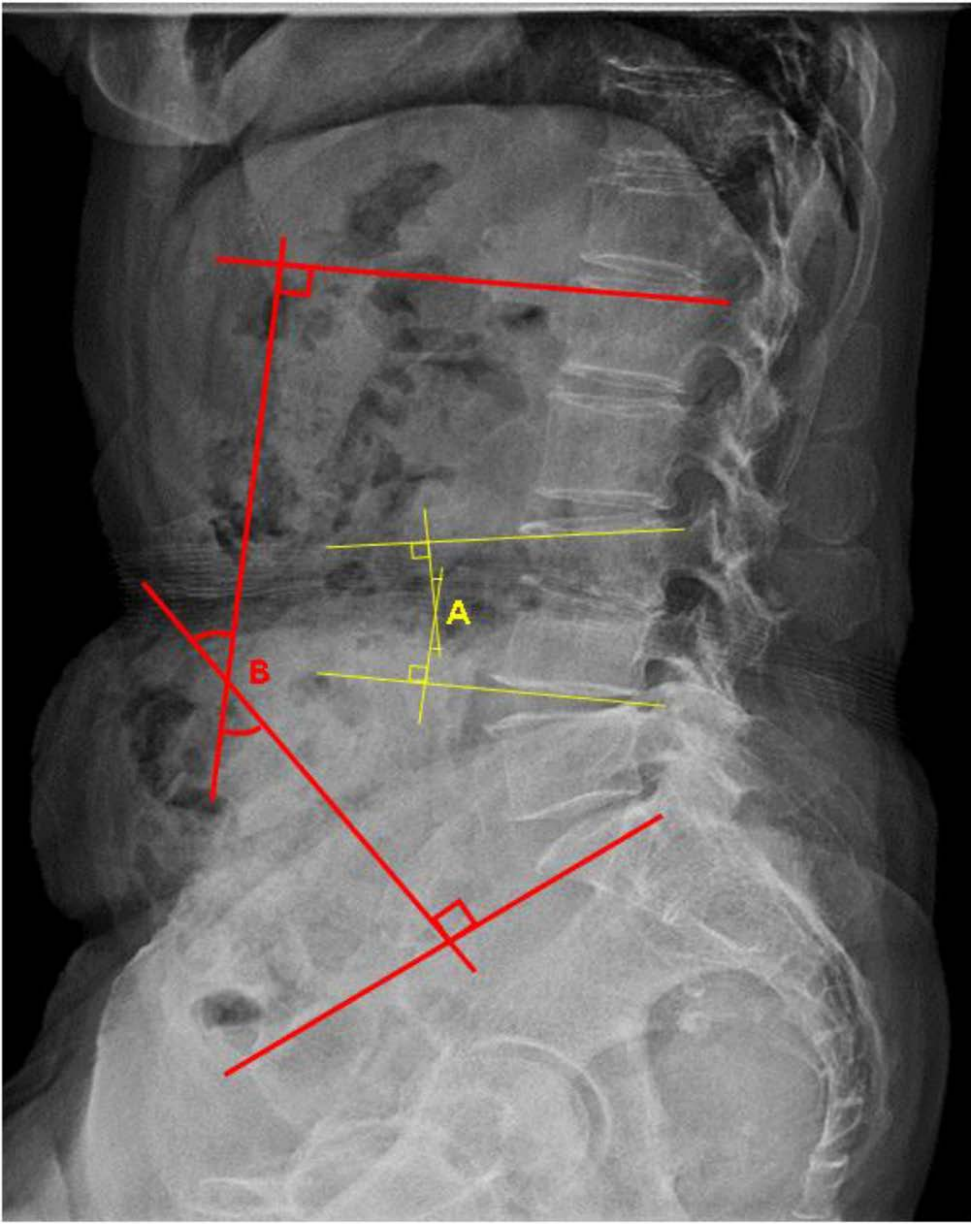
Measurement of the regional kyphotic angle (A) and the lordotic angle (B).

Patients’ demographic data (age, sex, segment of the lesion, American Society of Anesthesiologists scores, causative organisms, comorbidity), intraoperative data (intraoperative blood loss, operation time), clinical outcomes (VAS, success rate), laboratory markers (WBC count, ESR, CRP), and radiological parameters (regional kyphotic angle, lordotic angle) were reviewed. The regional kyphotic angle was measured between the upper and lower endplates of the affected segment. The lordotic angle was measured between the superior endplates of L1 and S1 (Fig. 1).

Clinical outcomes, laboratory markers, and radiological parameters were checked during the follow-up period. Treatment success was defined as normalization of CRP (<1.0 mg/dL) at 3 months postoperatively and at the last follow-up.^[15]^ Bone union was assessed using radiographs or computed tomography at the last follow-up. Treatment success was defined as normalization of CRP (<1.0 mg/dL) at 3 months postoperatively and at the final follow-up. Radiologic parameters were not included in this definition because residual bone destruction or alignment change could persist despite infection control, especially in the posterior-only fixation group. Although pain intensity was evaluated using the VAS score, it was not considered an objective criterion for defining infection resolution.

### 2.4. Statistical analysis

Continuous variables were analyzed using the independent *t*-test or Mann–Whitney *U* test for between-group comparisons. Additionally, two-way repeated-measures ANOVA with Bonferroni correction was used to examine the main effects of time and group, as well as the interaction between time and group, for the serial parameters. Categorical variables were assessed using the chi-square test or Fisher exact test, as appropriate. Statistical significance was defined as a *P*-value < .05. All analyses were performed using SPSS software (version 25.0; IBM Corp., Armonk).

## 3. Results

The mean age at the time of operation was 68.1 ± 12.1 years in the conventional group and 64.4 ± 11.7 years in the internal orthosis group (*P* = .302). The mean follow-up duration was 24.8 ± 23.1 months and 26.5 ± 18.8 months, respectively (*P* = .783). Baseline characteristics, including American Society of Anesthesiologists grade, causative organisms, and comorbidities, were comparable between the 2 groups (Table 1).

**Table 1:** Comparison of demographic characteristics.

Variable	Conventional group (n = 22)	Internal orthosis group (n = 23)	*P*-value
Sex (Male/Female)	12/10	15/8	.540
Age (yr)	68.1 ± 12.1	64.4 ± 11.7	.302
F/U duration (mo)	24.8 ± 23.1	26.5 ± 18.8	.783
ASA grade			
1	2	2	.523
2	17	15	
3	3	4	
4	0	2	
Organism			
*MSSA*	4	3	.639
*MRSA*	1	3	
*MRSE*	2	0	
*Streptococcus agalactiae*	2	2	
*Escherichia coli*	1	2	
*Staphylococcus epidemidis*	0	1	
*Klebsiella pneumoniae*	2	1	
*Enterococcus feacalis*	0	1	
*Haemophilus aphrophilus*	0	1	
*Klebsiella oxytoca*	0	1	
*Achromobacter denitrificans*	0	1	
*Bacteroides fragilis*	1	0	
*Citrobacter freundii*	1	0	
*Campylobacter fetus*	1	0	
*Mycobacterium tuberculosis*	1	0	
*No growth*	6	7	
Comorbidity			
Hypertension	11	7	.700
Diabetes mellitus	7	9	
Chronic kidney disease	0	1	
COPD	2	0	
Rheumatoid arthritis	1	1	
Liver cirrhosis	0	1	
Hypothyroidism	0	1	
Other infection	2	6	
Cardiology	3	1	
Neurology	2	2	
Hematology	0	1	
Oncology	4	3	

ASA = American Society of Anesthesiologists, COPD = chronic obstructive pulmonary disease, F/U = follow-up, MRSA = methicillin-resistant *Staphylococcus aureus*, MRSE = methicillin-resistant *Staphylococcus epidermidis*, MSSA = methicillin-sensitive *Staphylococcus aureus*.

The mean preoperative VAS score was 8 (7–9) in the conventional group and 8 (7–10) in the internal orthosis group (*P* = .448). The mean WBC count was 10.44 ± 4.41 × 10^3^/µL in the conventional group and 8.49 ± 3.25 × 10^3^/µL in the internal orthosis group (*P* = .098). The mean ESR values were 69.29 ± 31.87 mm/h and 86.14 ± 32.51 mm/h, respectively (*P* = .094), and the mean CRP levels were 8.65 ± 16.11 mg/dL and 6.28 ± 3.88 mg/dL, respectively (*P* = .516).

The median number of infected levels was 2 (1–3) in the conventional group and 2 (1–4) in the internal orthosis group (*P* = .958). The incidence of epidural and paravertebral abscesses was comparable between the 2 groups. Epidural abscess was identified in 7 patients (31.8%) in the conventional group and 12 patients (52.2%) in the internal orthosis group (*P* = .167). Paravertebral abscess was present in 16 patients (72.7%) and 18 patients (78.3%), respectively (*P* = .666). The mean preoperative regional kyphotic angle was 0.58 ± 17.16° in the conventional group and 6.55 ± 11.49° in the internal orthosis group (*P* = .181), and the mean lordotic angle was 13.40 ± 14.81° and 15.80 ± 11.37°, respectively (*P* = .551). None of these differences was statistically significant (Table 2).

**Table 2:** Comparison of preoperative parameters.

Variable	Conventional group (n = 22)	Internal orthosis group (n = 23)	*P*-value
Clinical parameter			
VAS (scale)	8 (7-9)	8 (7–10)	.448
Laboratory parameter			
WBC count (10^3^/µL)	10.44 ± 4.41	8.49 ± 3.25	.098
ESR (mm/h)	69.29 ± 31.87	86.14 ± 32.51	.094
CRP (mg/dL)	8.65 ± 16.11	6.28 ± 3.88	.516
Radiological parameter			
Infected segment (number of levels)	2 (1–3)	2 (1–4)	.958
Epidural abscess (n, %)	7 (31.8%)	12 (52.2%)	.167
Paravertebral abscess (n, %)	16 (72.7%)	18 (78.3%)	.666
Regional kyphotic angle (°)	0.58 ± 17.16	6.55 ± 11.49	.181
Lordotic angle (°)	13.40 ± 14.81	15.80 ± 11.37	.551

CRP = C-reactive protein, ESR = erythrocyte sedimentation rate, VAS = visual analogue scale, WBC = white blood cell.

Regarding intraoperative data, significant differences were observed in operative time and intraoperative blood loss. The mean operative time was 375.9 ± 125.5 minutes in the conventional group and 202.0 ± 71.2 minutes in the internal orthosis group (*P* < .001). Mean intraoperative blood loss was 645.5 ± 459.5 mL and 267.4 ± 122.1 mL, respectively (*P* < .001; Table 3).

**Table 3:** Comparison of intraoperative data.

Variable	Conventional group (n = 22)	Internal orthosis group (n = 23)	*P*-value
Operation time (min)	375.9 ± 125.5	202 ± 71.2	<.001
Intraoperative blood loss (mL)	645.5 ± 459.5	267.4 ± 122.1	<.001

**Figure 2.**
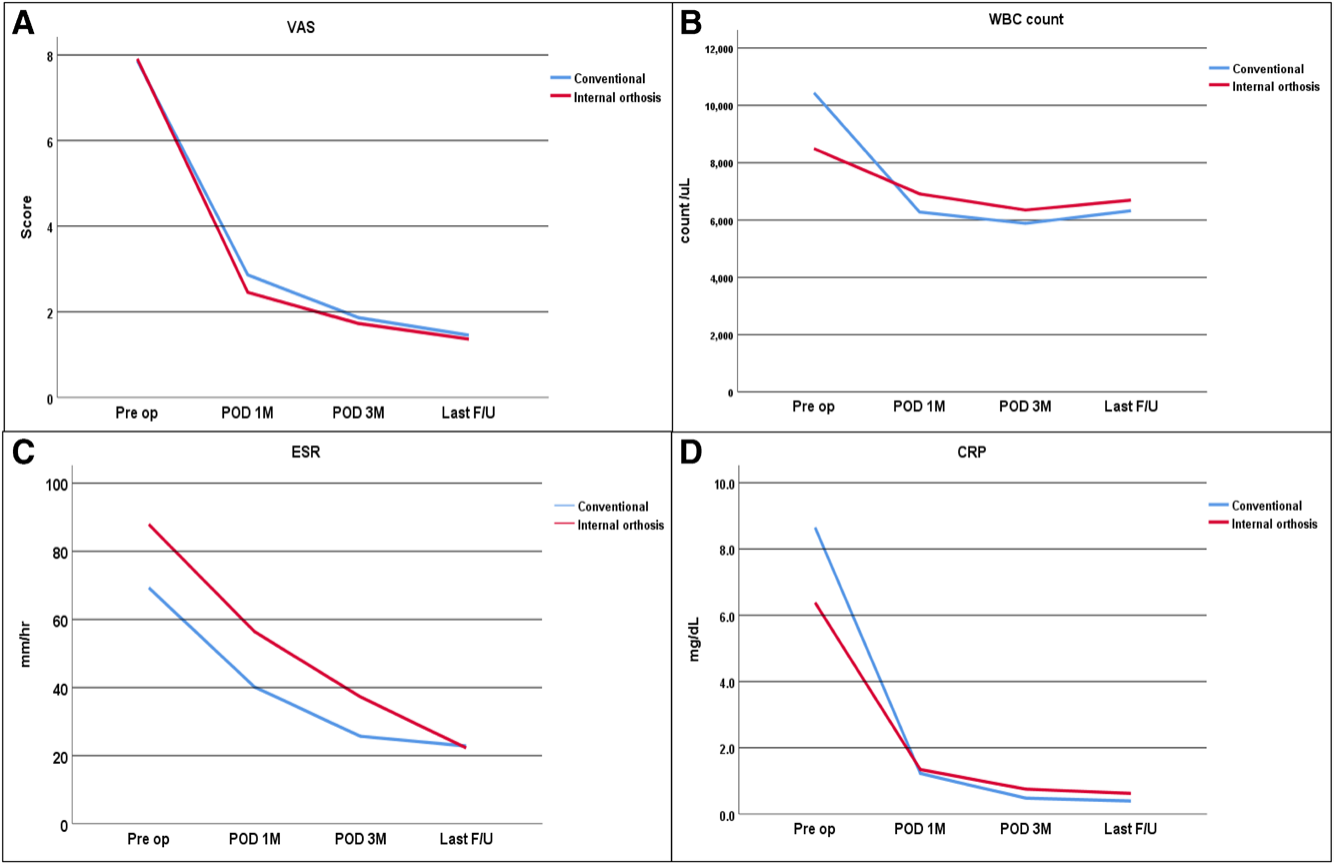
Graph of VAS score change (A), WBC count change (B), ESR change (C), and CRP change (D) postoperatively. CRP = C-reactive protein, ESR = erythrocyte sedimentation rate, POD = postoperative day, VAS = visual analog scale, WBC = white blood cell count.

There was no significant difference in the duration of postoperative antibiotic therapy (64.6 ± 67.8 days vs 71.4 ± 49.5 days, *P* = .454). For clinical outcomes and laboratory parameters, both groups showed gradual improvement in VAS, WBC, ESR, and CRP levels over time (Fig. 2). At one month postoperatively, the ESR was significantly lower in the conventional group than in the internal orthosis group (*P* = .028), although no significant difference was observed thereafter. At 3 months postoperatively, the treatment success rates were 90.9% in the conventional group and 91.3% in the internal orthosis group (*P* = 1.000). At the final follow-up, the rates were 95.5% and 87.0%, respectively (*P* = .608), showing no significant difference in treatment success between groups (Table 4).

**Table 4:** Comparison of postoperative clinical data.

Variable	Conventional group (n = 22)	Internal orthosis group (n = 23)	*P*-value
Postoperative antibiotics duration (d)	64.6 ± 67.8	71.4 ± 49.5	.454
POD 1 mo			
VAS (scale)	3 (1–5)	3 (1–4)	.169
WBC count (10^3^/µL)	6.28 ± 1.34	6.91 ± 2.16	.244
ESR (mm/h)	40.18 ± 17.70	56.20 ± 27.15	**.028**
CRP (mg/dL)	1.26 ± 1.54	1.74 ± 2.52	.635
POD 3 mo			
VAS (scale)	2 (1–4)	1 (0–4)	.941
WBC count (10^3^/µL)	5.88 ± 1.39	6.35 ± 1.71	.323
ESR (mm/h)	25.68 ± 16.17	37.26 ± 23.30	.069
CRP (mg/dL)	0.48 ± 1.11	0.72 ± 1.74	.593
Success rate	90.91%	91.30%	1.000
Last follow up			
VAS (scale)	1 (0–6)	1 (0–4)	.908
WBC count (10^3^/µL)	6.32 ± 1.84	6.69 ± 1.51	.469
ESR (mm/h)	22.82 ± 16.34	25.53 ± 16.97	.597
CRP (mg/dL)	0.40 ± 1.04	0.71 ± 1.53	.434
Success rate	95.45%	86.96%	.608

CRP = C-reactive protein, ESR = erythrocyte sedimentation rate, POD = postoperative day, VAS = visual analogue scale, WBC = white blood cell.

The mean regional kyphotic angles of the conventional and internal orthosis groups were −7.5 ± 12.8° and −2.9 ± 12.0° immediately after surgery (*P* = .228), −5.7 ± 12.1° and 2.0 ± 10.9° at one month postoperatively (*P* = .031), and − 2.7 ± 14.8° and 4.7 ± 11.7° at the final follow-up (*P* = .071). The corresponding mean lordotic angles were 20.0 ± 12.2° and 26.2 ± 12.4° immediately after surgery (*P* = .106), 15.1 ± 13.1° and 19.9 ± 12.3° at one month (*P* = .220), and 13.3 ± 16.6° and 17.3 ± 13.6° at the final follow-up (*P* = .380). Although the internal orthosis group tended to exhibit greater angular deformity (larger kyphotic and lordotic angles), these differences were not statistically significant except for the regional kyphotic angle at one month, which was significantly higher in the internal orthosis group (*P* = .031). At the final follow-up, the bone union rate was markedly higher in the conventional group (86.4%) than in the internal orthosis group (43.5%, *P* = .003; Table 5).

**Table 5:** Comparison of postoperative radiological data.

Variable	Conventional group (n = 22)	Internal orthosis group (n = 23)	*P*-value
Immediate postoperatively			
Regional kyphotic angle (°)	−7.52 ± 12.83	−2.94 ± 11.96	.228
Lordotic angle (°)	20.00 ± 12.15	26.18 ± 12.41	.106
POD 1 mo			
Regional kyphotic angle (°)	−5.71 ± 12.10	2.02 ± 10.85	.031
Lordotic angle (°)	15.05 ± 13.09	19.87 ± 12.29	.220
Last follow up			
Regional kyphotic angle (°)	−2.70 ± 14.78	4.74 ± 11.66	.071
Lordotic angle (°)	13.28 ± 16.55	17.34 ± 13.60	.380
Bone union (yes/no) (union rate)	19/3 (86.36%)	10/13 (43.48%)	.003

POD = postoperative day.

The results of two-way repeated-measures ANOVA are summarized in Table 6. Significant main effects of time were observed for all parameters (*P* < .001), indicating improvement or change over the postoperative period. The main effect of group was significant only for ESR (*P* = .034), with the conventional group showing lower ESR levels overall. A significant time*group interaction was found for WBC (*P* = .009), indicating a slightly faster decline in the conventional group. No other parameters demonstrated significant group or interaction effects.

**Table 6:** Two-way repeated-measures ANOVA.

Effect	*F* (df_1_, df_2_)	*P*-value	Partial η^2^	Observed power
VAS				
Time	694.29 (3, 41)	<.001	0.942	1.000
Group	0.65 (1, 43)	.426	0.015	0.123
Time*Group	1.17 (3, 41)	.326	0.026	0.307
WBC				
Time	25.56 (3, 41)	<.001	0.373	1.000
Group	0.06 (1, 43)	.808	0.001	0.057
Time*Group	4.02 (3, 41)	.009	0.085	0.830
ESR				
Time	54.21 (3, 38)	<.001	0.575	1.000
Group	4.80 (1, 40)	.034	0.107	0.571
Time*Group	1.41 (3, 38)	.244	0.034	0.366
CRP				
Time	12.20 (3, 38)	<.001	0.234	1.000
Group	0.09 (1, 40)	.760	0.002	0.060
Time*Group	0.52 (3, 38)	.668	0.013	0.154
Regional kyphotic angle				
Time	34.68 (3, 41)	<.001	0.446	1.000
Group	3.54 (1, 43)	.067	0.076	0.452
Time*Group	1.17 (3, 41)	.324	0.026	0.308
Lordotic angle				
Time	19.88 (3, 40)	<.001	0.321	1.000
Group	1.53 (1, 42)	.223	0.035	0.227
Time*Group	0.76 (3, 40)	.521	0.018	0.208

Partial η^2^ represents effect size; observed power indicates statistical power of each effect.

CRP = C-reactive protein, ESR = erythrocyte sedimentation rate, VAS = visual analog scale, WBC = white blood cell count.

* Two-way repeated-measures ANOVA with Bonferroni correction.

## 4. Discussion

The present study aimed to evaluate the clinical and radiologic outcomes of posterior percutaneous fixation without anterior or posterior debridement (“internal orthosis”) compared with conventional anterior debridement and posterior fixation in patients with infectious spondylitis. Both surgical methods achieved satisfactory infection control and pain relief, while posterior-only fixation significantly reduced operative time and intraoperative blood loss. Although the rate of radiologic bone union was lower in the posterior-only group, the overall clinical success rate was comparable between the 2 techniques, suggesting that posterior percutaneous fixation without debridement can serve as a less invasive and effective surgical alternative for patients with high morbidity.

Bhavuk et al reported better neurologic recovery and functional outcomes in the anterior group than in the posterior group, but poorer correction of kyphotic deformity in the anterior group.^[10]^ Choi et al and An et al reported lumbar interbody fusion with bone graft through the posterior approach and showed good results and low morbidity.^[13,16]^ These are similar to this study in that only the posterior approach was used, but in this study, only percutaneous pedicle screw fixation was performed without interbody fusion.

Few studies have investigated percutaneous pedicle screw fixation-only (i.e., without anterior debridement) for infectious spondylitis. Nasto et al reported that percutaneous posterior screw fixation is a safe and effective procedure for pain relief and prevents deformity but does not improve healing time.^[17]^ Deininger et al reported good clinical outcomes with percutaneous posterior screw fixation in infectious spondylitis.^[18]^ Gamada et al reported the result of minimally invasive posterior fixation without anterior lesion debridement or bone grafting for thoracolumbar pyogenic spondylitis. In their study, 31 patients underwent surgery, and 4 patients (14%) required additional surgeries for infection control.^[19]^ Their result is similar to our success rate (86.96%) at the last follow-up in the internal orthosis group. The present study is noteworthy in that it compared internal orthosis to the conventional procedure and produced satisfactory results despite being more minimally invasive.

In the present study, inflammatory markers, including ESR and CRP, showed a steady decline after surgery in both groups, reflecting effective infection control. The ESR tended to decrease more rapidly in the conventional group during the early postoperative period, although both surgical methods ultimately achieved comparable normalization of inflammatory markers over time. These findings are consistent with previous reports indicating that minimally invasive posterior fixation provides adequate infection control with reduced surgical morbidity.^[17–19]^

Radiologic outcomes demonstrated that, during the early postoperative period, the internal orthosis group exhibited more pronounced kyphotic deformity compared with the conventional group. This difference is likely attributable to the lack of anterior column reconstruction in the posterior-only fixation technique. In contrast, the conventional approach included bone grafting or cage insertion that directly supported the affected segments. However, these early differences did not persist at the final follow-up, suggesting that spinal alignment was adequately maintained over time in both groups. Notably, the posterior-only approach achieved these outcomes with significantly less operative time and blood loss, supporting its role as a less invasive yet effective alternative to the anterior–posterior combined procedure for infectious spondylitis.

The results of the two-way repeated-measures ANOVA provide further validation of these findings. All clinical and laboratory parameters, including VAS, WBC, ESR, and CRP, demonstrated significant improvement over time, confirming consistent postoperative recovery in both groups. The absence of significant group effects for most parameters indicates that posterior-only fixation achieved clinical outcomes equivalent to those of conventional anterior debridement and posterior fixation. Although the interaction between time and group reached significance only for WBC, this likely reflects a transient postoperative inflammatory response rather than a clinically meaningful difference. Radiologic parameters, including kyphotic and lordotic angles, also changed significantly over time but showed no group-dependent differences, supporting the concept that spinal alignment was adequately maintained with the posterior-only fixation technique.

There was a significant difference in the bone union rate at the final follow-up between the 2 groups. This finding is expected, as the primary goal of posterior percutaneous fixation (“internal orthosis”) is to stabilize the infected segment rather than to promote interbody fusion. Nevertheless, spontaneous bone union was observed in several patients in the internal orthosis group, indicating that solid fusion can occasionally occur even without anterior reconstruction. Masaki et al reported a case of spontaneous bone regeneration and interbody fusion after percutaneous pedicle screw fixation for intractable pyogenic spondylitis with a large vertebral defect.^[20]^ They proposed that residual bony scaffolds may persist even in decalcified vertebrae and can undergo remineralization once infection control is achieved. This hypothesis may also explain the spontaneous fusion cases observed in the present study, supporting the potential for biological recovery of vertebral integrity following eradication of infection and stable fixation.

This study has several limitations. First, it was conducted at a single center with a relatively small sample size, which may limit the generalizability of the findings. Second, its retrospective design may introduce selection bias, and multivariable analyses were not performed to adjust for potential confounding factors such as baseline infection severity or comorbidities. Third, the follow-up duration was relatively short, which may have underestimated the incidence of late complications or recurrence. Fourth, treatment success was defined solely by CRP normalization, which, although objective, may not fully reflect functional recovery or radiologic improvement. Future multicenter prospective studies with larger cohorts, longer follow-up, and comprehensive multivariate analyses are needed to validate and expand upon these findings.

## 5. Conclusion

Internal orthosis can be selectively performed to treat infectious spondylitis as an alternative to the conventional procedure if the risk of surgical treatment is high due to the poor general condition of patients.

## Acknowledgments

This study was financially supported by Chonnam National University (Grant number: 2025-0367-01).

## Author contributions

**Conceptualization:** Sung-Kyu Kim, Hyoung-Yeon Seo.

**Data curation:** Chan Young Lee, Hyoung-Yeon Seo.

**Formal analysis:** Chan Young Lee.

**Investigation:** Sung-Kyu Kim.

**Methodology:** Chan Young Lee.

**Resources:** Chan Young Lee.

**Supervision:** Hyoung-Yeon Seo.

**Validation:** Chan Young Lee.

**Visualization:** Chan Young Lee.

**Writing – original draft:** Chan Young Lee, Hyoung-Yeon Seo.

**Writing – review & editing:** Chan Young Lee, Sung-Kyu Kim, Hyoung-Yeon Seo.
